# Culture supernatant of adipose stem cells can ameliorate allergic airway inflammation via recruitment of CD4^+^CD25^+^Foxp3 T cells

**DOI:** 10.1186/s13287-016-0462-5

**Published:** 2017-01-23

**Authors:** Hak Sun Yu, Mi-Kyung Park, Shin Ae Kang, Kyu-Sup Cho, Sue Jean Mun, Hwan-Jung Roh

**Affiliations:** 1 Department of Parasitology, Pusan National University School of Medicine, Yangsan, 626-870 Republic of Korea; 2 Department of Otorhinolaryngology and Biomedical Research Institute, Pusan National University Hospital, Busan, 602-739 Republic of Korea; 3 Department of Otorhinolaryngology and Research Institute for Convergence of Biomedical Science and Technology, Pusan National University Yangsan Hospital, Beomeo-li, Mulgeum-eup, Yangsan-si, Gyeongsangnam-do, Yangsan, 626-770 Republic of Korea; 4 Immunoregulatory Therapeutics Group in Brain Busan 21 project, Yangsan, Republic of Korea

**Keywords:** Adipose stem cell, Supernatant, Allergic airway inflammation, Treg cell

## Abstract

**Background:**

In a previous study, we demonstrated that intravenous administration of adipose tissue stem cells (ASCs) could significantly reduce allergic symptoms and suppress eosinophilic inflammation.

**Methods:**

To evaluate the secretome of ASCs, we administrated culture supernatant of ASCs (ASC sup, which contains the ASC secretome) and uncultured fresh medium (con sup) into a mouse model of allergic airway inflammation. Subsequently we observed the mice for signs of inflammation and investigated Th1-, Th2-, and T_reg_-related cytokine levels as well as recruitment of T_reg_ cells into the airway.

**Results:**

We found that ASC sup could ameliorate allergic airway inflammation in this model; the value of airway hyperresponsiveness, and the occurrence of inflammatory cell infiltration in the lung, as well as the number of eosinophils, and goblet cells in the lung epithelium were all significantly decreased by ASC sup treatment. In addition, ASC sup treatment significantly decreased the levels of IL-4, IL-5, and IL-13 in the bronchial alveolar lavage fluid and in culture medium of lung-draining lymph node cells of the animal model of acute asthma. We detected numerous CTLA-4 and Foxp3-expressing cells in the lung after ASC sup treatment. ASC sup was found to have a higher concentration of IL-10 and TGF-β compared to con sup.

**Conclusions:**

Stem cells have powerful potential for therapeutic functions in various diseases, but they also have many drawbacks. In this study, we found strong immunosuppressive ability of ASC sup in an allergic airway mouse model. It may be possible to use ASC sup for treatment of many immunological diseases in the near future.

**Electronic supplementary material:**

The online version of this article (doi:10.1186/s13287-016-0462-5) contains supplementary material, which is available to authorized users.

## Background

Mesenchymal stem cells (MSCs) have been isolated from a variety of tissues, such as skeletal muscle, bone marrow, chondrocytes, umbilical cord, bone, and adipose tissue [1]. The expansive abilities of MSCs make them one of the most important candidates for disease therapy and regenerative medicine. In addition, they have strong immunosuppressive activity in different situations, and clinical trials are ongoing evaluating them for treatment of immunological disease [2–4]. MSCs could control the immune function of most immune cells involved in allergen and antigen recognition of antigen-presenting cells, natural killer cells, T cells, and B cells [2].

MSCs derived from adipose tissue stem cells (ASCs) may share with other MSCs the ability to suppress inflammation and immune responses [5]. Our research group is interested in the possibility of using ASCs as therapeutic agents for allergic airway diseases. We demonstrated that intravenous administration of ASCs could reduce allergic symptoms significantly and suppress eosinophilic inflammation [6, 7]. In addition, we found that ASCs significantly suppressed the production of Th2-associated cytokines [interleukin (IL)-4, IL-5, and IL-13], and improved Th1 cytokine [interferon gamma (IFN-γ)] production, in a mouse model of allergic airway inflammation. In addition, CD4^+^CD25^+^Foxp3^+^ T cells (regulatory T cells, T_reg_) and regulatory cytokines [IL-10 and transforming growth factor-beta (TGF-β)] were significantly increased in the lung immune system [6, 7]. Moreover, this activation was inhibited by prostaglandin E2 (PGE2) and TGF-β-neutralizing antibodies. Because PGE2 and TGE-β play a role in inducing T_reg_ expansion, the immune suppression effects of ASCs are closely related with T_reg_ cell recruitment and activation.

Recently, it has been reported that culture supernatant of MSCs could suppress T cell proliferation in an in vitro model [8, 9], and that feline mesenchymal stem cell supernatant could inhibit reactive oxygen species production by feline neutrophils [10]. Cruz et al., suggested that human bone marrow-derived cells extracellular vesicles also ameliorate *Aspergillus* hyphal extract-induced allergic airway inflammation in immunocompetent mice [11]. In addition, Ionescu et al., reported that secreting soluble factors of bone marrow-derived cell prevented airway hyperresponsiveness (AHR) and inflammation. In the chronic asthma model, the soluble factors prevented airway smooth muscle thickening and peribronchial inflammation [12]. The soluble factors upregulated an IL-10-induced and IL-10-secreting subset of T regulatory lymphocytes and promoted IL-10 expression by lung macrophages [12]. However, there are no reports on whether secreted soluble factors of human ASCs can act as an anti-inflammatory and immune-regulatory response under airway inflammation situations like those of bone marrow-derived cells.

Lee et al. reported the secretion of 187 proteins from human ASCs activated by tumor necrosis factor-alpha (TNF-α) [13]. Therefore, we reasoned that ASCs could secrete many proteins (secretome) including cytokines and chemokines in an artificial culture system; this secretome might be a good candidate for immunoregulatory therapeutic agents. In this study, we administrated culture supernatant of ASCs (ASC sup) to a mouse model of allergic airway inflammation, and observed their signs of airway inflammation. We also investigated Th1-, Th2-, and T_reg_-related cytokine levels and recruitment of T_reg_ cells to the airway. Additionally we studied the expression level of chemokine genes in mouse lung epithelial cells after stimulation with ASC sup.

## Methods

### Animals

Six-week-old female C57BL/6 mice were purchased from Samtako Co. (Osan, Republic of Korea), and Foxp3-GFP (expressing GFP-tagged Foxp3) mice were purchased from the Jackson Laboratory, Bar Harbor, ME, USA. They were bred in a specific pathogen-free animal facility during experiments. The animal study protocol was approved by the Institutional Animal Care and Use Committee of the Pusan National University (Approval No. PNU-2016-1109).

### Isolation and culture of ASCs

Adipose tissue was obtained from the abdominal fat of C57BL/6 mice according to previous reports [6, 14]. Briefly, adipose tissue was digested with 0.075% collagenase type I (Sigma-Aldrich, St. Louis, MO, USA) at 37 °C for 30 min after washing with phosphate-buffered saline (PBS). After neutralization, the sample was centrifuged at 1200 × *g* for 10 min. The pellet was incubated overnight at 37 °C in 5% CO_2_ in control medium [α-MEM, 10% fetal bovine serum (FBS), 100 unit/ml penicillin, 100 μg/ml streptomycin]. Following incubation, residual non-adherent cells were removed. The attached cells of ASCs (third or fourth passages) were used in experiments after phenotypic classification of the ASCs, according to previous methods [6, 14].

### ASC sup collection and endotoxin depletion

ASCs, at a concentration of 1 × 10^5^ cells/cm^2^, were cultured until reaching 1 × 10^6^ cells/cm^2^ (about 48 hours) in α-MEM containing 10% FBS at 37 °C in 5% CO_2_ [6]. After centrifugation (12,000 × *g* for 30 min), the supernatants of ASC culture (ASC sup) and fresh culture medium control supernatant (con sup) were collected and concentrated (about 50- fold) by applied pressure using a concentrator (Amicon, Millipore Corporations, Billerica, MA, USA) with 3000-Da pore size membranes. The unnecessary excessive salts were eliminated from collected supernatants using a HiTrap Desalting™ kit (GE Healthcare, Uppsala, Sweden). Lipopolysacharide (LPS) was depleted (endotoxin levels < 0.01 μg/ml) from the concentrated supernatant using Detoxi-Gel Affinity Pak prepacked columns (Pierce, Rockford, IL, USA), in accordance with the manufacturer’s instructions.

### Mouse model of allergic airway inflammation

**Fig. 1:**
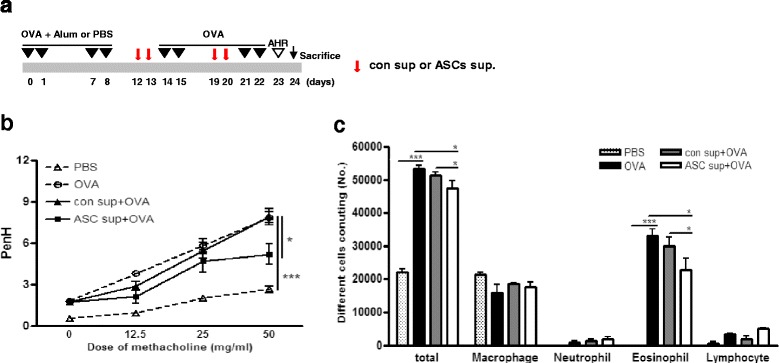
The adipose-derived stem cell cultured supernatant (ASC sup) reduced OVA-Alum-induced allergic airway inflammation. **a** Mice were sensitized on days 0, 1, 7, and 8 by intraperitoneal injection of OVA plus Alum or PBS (control) only. The mice were challenged on days 14, 15, 21, and 22 intranasally with OVA. 10 μg/50 μL of the con sup or ASC sup were injected intranasally on days 12, 13, 19, and 20. **b** The enhanced pause (PenH) was evaluated at baseline and after treatment with increasing doses of aerosolized methacholine (0–50 mg/ml). *Open triangle*; PBS-treated mice [*PBS*], *open circle*; Ova-Alum-treated mice [*OVA*], *solid triangle*; control sup-treated mice after Ova-Alum treatment [*con sup + OVA*], *solid square*; ASC sup-treated mice after Ova-Alum treatment [*ASC sup + OVA*]. **b** Airway resistance values in response to methacholine (0 to 50 mg/ml) were evaluated. **c** The number of inflammatory cells in the BALF samples was counted after Diff-Quik staining. (^*^ *p* < 0.05, ^***^ *p* < 0.001, n = 5 mice per group, experiments were performed in triplicate)

A mouse model of allergic airway inflammation was induced as previously reported with minor modification [14, 15]. Briefly, mice were sensitized by intraperitoneal injection of 75 μg of OVA (Sigma-Aldrich, St. Louis, MO, USA) in 200 μL PBS containing 10 mg/ml aluminum hydroxide (Sigma-Aldrich) on days 0, 1, 7, and 8. On days 14, 15, 21, and 22 after the initial sensitization, the mice were challenged intranasally with 50 μg of OVA in 50 μL PBS (Fig. 1a).

### Measurement of airway hyperresponsiveness (AHR)

Twenty-four hours after the last challenge, the AHR was assessed in conscious, unrestrained mice using noninvasive whole-body plethysmography (Allmedicus, Seoul, Korea) as previously described [15]. In brief, the mice were placed in the plethysmography chamber and exposed to increasing concentrations of aerosolized methacholine at 0, 12.5, 25, and 50 mg/ml for 10 min. The enhanced pause (PenH) was calculated automatically based on the mean pressure generated in the plethysmography chamber during inspiration and expiration combined with the time of each phase. The PenH values calculated during each 3-min interval were then averaged.

### Differential cell counting in bronchoalveolar lavage fluid (BALF)

After sacrificing the animal, the BALF was collected from mouse lungs as outlined in previous reports [15, 16]. The BALF samples were centrifuged for 5 min at 500 × *g* at 4 °C. The supernatants were collected and immediately frozen at -70 °C. Cell pellets were resuspended and washed twice in PBS. The total cell numbers were counted using a hematocytometer. BALF cell smears were prepared using a cytospin apparatus, and stained with Diff-Quik solution (Sysmex Co., Kobe, Japan) to determine the differential cell counts in accordance with conventional morphological criteria. At least 500 cells per slide were evaluated in order to obtain the differential leukocyte counts.

### Lung histology and inflammation scoring

Lung tissues were removed after lavage, fixed in 10% neutral formalin for 36 hours, and embedded in paraffin. Thin sections of embedded tissues were stained with hematoxylin and eosin (H&E) for identification of eosinophils and periodic acid-Schiff (PAS) for counting mucin-secreting cells. The lung inflammation score was assessed by the degree of peribronchial and perivascular inflammation, which were evaluated on a subjective scale of 0–4 as previously described [17]. For quantifying goblet cell hyperplasia, the percentage of PAS-positive cells in epithelial areas was examined in eight to ten tissue sections per mouse.

### Measurement of serum immunoglobulins

At 48 hours after the last OVA challenge, serum was collected from mice via cardiac puncture. Total and OVA-specific immunoglobulins (IgE, IgG1, IgG2a) were determined by enzyme-linked immunosorbent assay (ELISA) according to the manufacturer’s instructions (R&D Systems, Minneapolis, MN, USA). Absorbance (450 nm) was measured with an ELISA plate reader (Molecular Devices, Sunnyvale, CA, USA).

### Expression of cytokines in the BALF and lung-draining lymph nodes

Lung-draining lymph nodes (LLNs) were observed between the trachea and both lung lobes in the OVA-induced animal model of acute asthma. Lymphocytes were isolated from LLNs according to previous reports [14]. The lymphocytes were plated in 48-well plates coated with 0.5 μg/ml CD3 antibody (BD Pharmigen™, BD Biosciences, San Jose, CA, USA) at a concentration of 10^6^ cells/ml in RPMI 1640 with 10% FBS. Plated cells were incubated for 72 hours at 37 °C with 5% CO_2_. After stimulation, the supernatant was used for experiments. The concentrations of IL-4, IL-5, IL-10, IL-13, interferon (IFN)-γ, and TGF-β in the BALF and in supernatants of LLNs were examined using ELISA kits according to the manufacturer’s instructions (eBioscience, San Diego, CA, USA). The absorbance of the final reactant was determined at 450 nm with an ELISA plate reader.

### FACS analysis of T cell distribution in LLN

To evaluate the recruitment of Th1, Th2, and T_reg_ induced by ASC sup treatment, the LLN cells of the OVA-induced animal model of acute asthma and ASC sup-treated animal model of acute asthma were cultured on anti-CD3-coated plates for 6 hours. To determine the CD4^+^CD25^+^Foxp3^+^ (T_reg_) and IL-10^+^/CD4^+^ T cell populations, the cells were stained with anti-CD4-FITC (0.5 mg/ml) and/or anti-CD25-APC (0.2 mg/ml) in accordance with the manufacturer’s recommendations (eBioscience, San Diego, CA, USA). After surface staining, the cells were permeabilized using a Cytofix/Cytoperm kit (eBioscience). After permeabilization, the cells were stained with anti-Foxp3-PE-cy7 or anti-IL-10-PE (eBioscience). To quantify the Th1 and Th2 cell populations, the LLN cells were stained with an anti-CD4-FITC antibody. After surface staining, the CD4^+^ T cells were stained with intracellular anti-IFN-γ-PE-cy7 (eBioscience) and anti-IL-4-PE (eBioscience) antibodies. Fluorescence was measured using a FACS CantoII cytometer (BD Biosciences) equipped with Canto software (BD Biosciences).

### Determination of T_reg_ cell recruitment in the lung

To test T_reg_ cell recruitment to the lung after ASC sup treatment, allergic airway inflammation was induced in Foxp3-GFP mice treated with ASC sup or control sup. After induction, five mice per group were sacrificed and lung tissues were embedded in paraffin. Some sections were stained with an anti-CTLA-4 antibody (activation marker of T_reg_) as previously reported [15]. The Alexa Fluor 594 goat anti-hamster IgG secondary antibody (1∶500; Jackson ImmunoResearch Laboratories, West Grove, PA, USA) was applied to the slide for 1 hour at 24 °C. The slides were washed in PBS and incubated with DAPI for 2 min. Confocal images of stained lung tissue or stained T_reg_ cells were examined under an inverted fluorescence microscope.

### SDS-polyacrylamide gel electrophoresis (SDS-PAGE) and western blot

To identify the molecular weights of proteins in the supernatant samples, SDS-PAGE was performed using ASC sup and control sup, according to the manufacturer’s instruction Bio-Rad Laboratories, Hercules, CA, USA). The electrophoresed gels were transferred to Hybond-C extra nitrocellulose membranes (Amersham Biosciences, Little Chalfont, UK) as performed in a previous study [18]. TGF-β in the ASC sup was measured using an anti-TGF-β antibody (10 μg/g body weight in 200 μL PBS; R&D Systems, Minneapolis, MN, USA).

### Statistical analysis

All experiments were repeated a minimum of three times. Data are expressed as mean ± SEM. Statistical significance was assessed by the Student’s *t* test using the SPSS software package version 18.0 (SPSS Inc., Chicago, IL, USA). A value of *p* < 0.05 was considered significant.

## Results

### Treatment with ASC sup suppressed OVA-induced airway inflammation

**Fig. 2:**
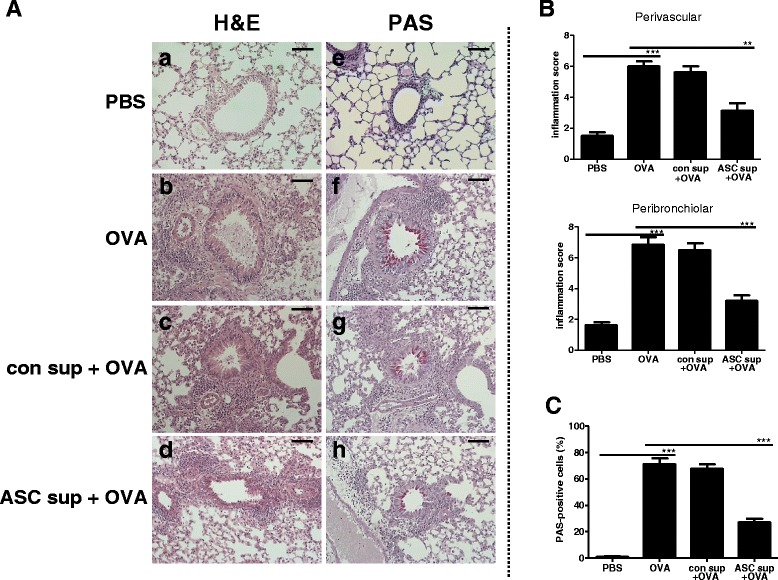
Treatment with ASC sup decreased inflammatory cell infiltration and mucus production. **a** Tissue inflammation observed in stained lung sections (*a*, *e*: PBS-treated mice [*PBS*]; *b*, *f*: OVA-Alum-induced airway inflammation mice [*OVA*]; *c*, *g*: OVA-Alum-induced airway inflammation mice with con sup treatment [*con sup + OVA*]; *d*, *h*: OVA-Alum-induced airway inflammation mice with ASC sup treatment [*ASC sup + OVA*]; *a*-*d*: H&E-stained; e-h: PAS-stained (bar = 100 μm). **b** Portions of hematoxylin-and eosin-stained inflated lung sections were blindly examined under light microscopy to assess the inflammation score of each section. We determined the inflammation score, which is the product of the *severity* and *prevalence* of inflammation, performed by as previous desribed [17]. *Severity* was assigned a numerical value based on the thickness of the inflammatory cell infiltrates surrounding the airways and blood vessels in the lung (0 = no cells; 1 = 1 − 3 cells thick; 2 = 4 − 6 cells thick; 3 = 7 − 9 cells thick; 4 = greater than or equal to 10 cells thick). *Prevalence* was assigned a numerical value, according to the percentage of airways and blood vessels in each section encompassed by inflammatory cells (0 = no airways or blood vessels; 1 = <25%; 2 = 25 − 50%; 3 = 51 − 75%; 4 = >75%). **c** The percentage of PAS-positive cells in epithelial areas was examined from eight to ten tissue sections per mouse. Each value is expressed as the mean ± SD. (^**^ *p* < 0.01, ^***^ *p* < 0.001, n = 5 mice per group, experiments were performed in triplicate)

To demonstrate the effects of ASC sup on OVA-induced airway inflammation, we pretreated the airways of an OVA-induced mouse model with ASC sup (at 10 μg/50 μl), and assessed biological and pathological changes (Fig. 1a). The value of AHR to methacholine was increased in OVA-induced mice. However, when treating with ASC sup, AHR was significantly diminished (Fig. 1b). In addition, control sup treatment also decreased the AHR value; however, the AHR values of control sup-treated mice were significantly higher than those of ASC sup-treated mice. Inflammatory cell infiltration was also observed in the airways after OVA-induced allergic inflammation. The numbers of eosinophils were especially increased after OVA induction of allergic airway inflammation. However, when mice were treated with ASC sup, eosinophil numbers significantly decreased in the airway (Fig. 1c). Histological examination of the lungs, after OVA-induced airway inflammation, exposed massive inflammatory cell infiltration, bronchial epithelial cell hyperplasia, and goblet cell hyperplasia (Fig. 2a). However, treatment with ASC sup remarkably decreased infiltration of inflammatory cells, goblet cell hyperplasia, and mucin production (Fig. 2a). Furthermore, after OVA treatment, the number of inflammatory cells was significantly lower around the perivascular and peribronchiolar regions of ASC sup-treated mice than control mice (Fig. 2b). In addition, the percentage of PAS-positive cells in the epithelial area of OVA-treated mice was up to 80%; however, only 30% PAS-positive cells were observed in the epithelia of the ASC sup-treated group (Fig. 2c).

### ASC sup treatment reduced OVA–induced Th2 response in the BALF and LLN

**Fig. 3:**
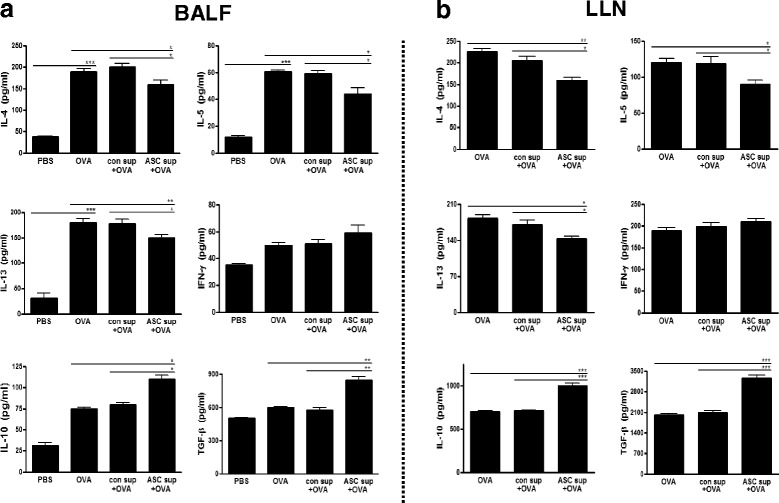
Treating with ASC sup regulated cytokine expression levels in OVA-induced BALF and LLNs. Cytokine concentrations in BALF(**a**) and in the culture medium of CD3-stimulated lymphocytes isolated from LLNs(**b**). For activation of lymphocytes from LLN, the wells were incubated with 1 μg/ml of anti-CD3 antibody for 16 hours at 4 °C. The lymphocytes (4 × 10^5^ cells per well) were introduced to the well and incubated for 3 days. After activation, the levels of several cytokines were measured in the supernatant using ELISA kits; ELISA assays were conducted in accordance with the manufacturer’s instructions. (*PBS;* BALF or culture supernatant of LLN from PBS-treated mice, *OVA;* BALF or culture supernatant of LLN from OVA-Alum-induced airway inflammation mice, *con sup + OVA;* BALF or culture supernatant of LLN from OVA-Alum-induced airway inflammation mice with con sup treatment, *ASC sup + OVA;* BALF or culture supernatant of LLN from OVA-Alum-induced airway inflammation mice with ASC sup treatment. ^*^ *p* < 0.05, ^**^ *p* < 0.01, ^***^ *p* < 0.001, n = 5 mice per group, experiments were performed in triplicate)

**Fig. 4:**
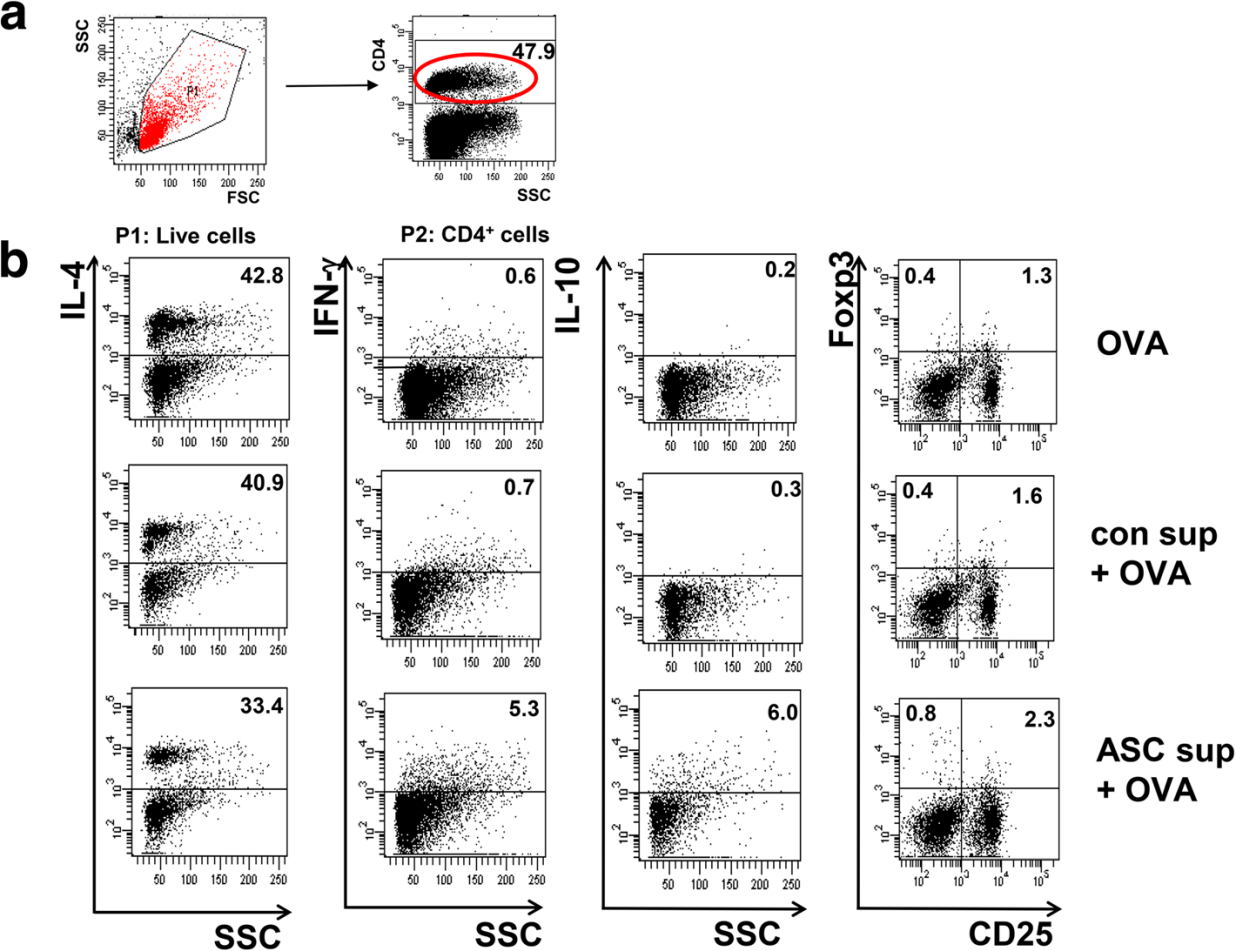
Regulation of T cell subsets by treatment with ASC sup. The lymphocytes from LLNs were cultured with stimulated anti-CD3 antibody. Each 1 × 10^6^ lymphocytes per sample were analyzed after surface and internal staining with various antibodies. After gating with CD4^+^ T cells (**a**), IL-4^+^, IFN-γ^+^, IL-10^+^, CD25^+^, and Foxp3^+^, T cells were counted by FACS analysis (**b**). (*OVA* lymphocytes from LLN of OVA-Alum-induced airway inflammation mice, *con sup + OVA* lymphocytes from LLN of OVA-Alum-induced airway inflammation mice with con sup treatment, *ASC sup + OVA* lymphocytes from LLN of OVA-Alum-induced airway inflammation mice with ASC sup treatment. n = 5 mice per group, experiments were performed in triplicate)

**Fig. 5:**
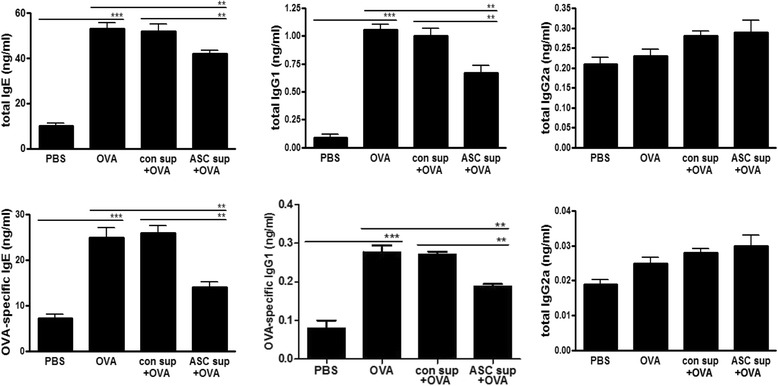
ASC sup treatment decreases total and OVA-specific IgE and IgG1 expression. The mice serum was diluted (as indicated) in PBS, for measurement of OVA-specific IgE (1:40), IgG1 (1: 10^5^), and IgG2a (1:10^5^) levels by ELISA. (*PBS;* serum from PBS-treated mice, *OVA*; serum from OVA-Alum-induced airway inflammation mice, *con sup + OVA;* serum from OVA-Alum-induced airway inflammation mice with con sup treatment, *ASC; sup + OVA* serum from OVA-Alum-induced airway inflammation mice with ASC sup treatment. ^*^ *p* < 0.05, ^**^ *p* < 0.01, ^***^ *p* < 0.001, n = 5 mice per group, experiments were performed in triplicate)

BALF and LLN lymphocytes from OVA-induced mice showed significantly increased levels of IL-4, IL-5, and IL-13 in the culture media of these cells (Fig. 3a and b). However, ASC sup treatment of the animal model of acute asthma in this model significantly decreased these high levels of IL-4, IL-5, and IL-13 (Fig. 3a and b). In contrast, ASC sup treatment significantly increased IL-10 and TGF-β in both samples from the animal model of acute asthma (Fig. 3a and b). Similar results were obtained from FACS analysis of LLN cells. IL-4-secreting CD4^+^ T cells were significantly decreased, but IFN-γ-secreting CD4^+^ T cells were considerably increased in the ASC sup-treated group compared to the OVA or OVA control sup group. The population of CD4^+^CD25^+^Foxp3^+^ T cells and IL-10-secreting CD4^+^ T cells were obviously increased by administration of ASC sup treatment in the animal model of acute asthma compared to those of the control sup-treated asthmatic group (Fig. 4). Also mean fluorescence intensity (MFI) of CD4^+^CD25^+^Foxp3^+^ markers were considerably increased in the ASC sup-treated group compared to the OVA or OVA control sup group (Additional file 1: Figure S1). Furthermore, total and OVA-specific IgE and IgG1 levels were significantly higher in the OVA group than in the PBS-treated group; however, intranasal pretreatment of ASC sup significantly decreased total IgE and OVA-specific IgE in the animal model of acute asthma (Fig. 5).

### Foxp3^+^ cells infiltrate the airway around sites of inflammation

**Fig. 6:**
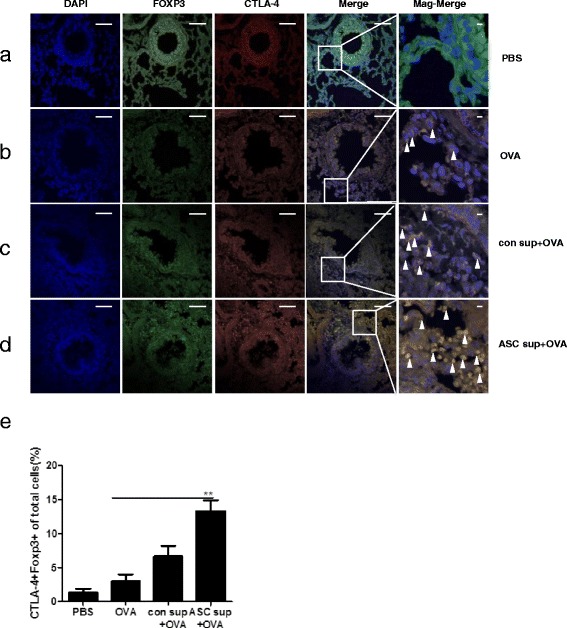
Recruitment of T_reg_ cells into the lung after ASC sup treatment. Paraffin sections of lungs from Foxp3-eGFP mice (*green*) were stained by immunofluorescence for CTLA-4 (*red*), and nuclei (DAPI, *blue*) representative pictures are shown, (*white bar* = 100 μm). (**a**) *PBS* lung of PBS-treated mice, (**b**) *OVA* lung of OVA-Alum-induced airway inflammation mice, (**c**) *con sup + OVA* lung of OVA-Alum-induced airway inflammation mice with con sup treatment, (**d**) *ASC sup + OVA* lung of OVA-Alum-induced airway inflammation mice with ASC sup treatment, *Merge*; merge of CTLA-4, GFP, and DAPI fields, *Mag-Merge;* magnification of Merge, *Arrowhead*; indicated CTLA4^+^Foxp3^+^ T cells. (**e**) Average ratio of CTLA4^+^Foxp3^+^ cells among total cells in the lung of each group was analyzed using the Image J program. (The value was calculated by three different authors who analyzed ten random spots of each slides). n = 5 mice per group, experiments were performed in triplicate

To evaluate T_reg_ cell migration to lung, we also induced allergic airway inflammation (using OVA) in Foxp3-eGFP mice after pretreatment with control sup and ASC sup as above. GFP-expressing Foxp3 cells were observed using confocal microscopy. After induction of allergic airway inflammation, some Foxp3-expressing cells were found in the lung matrix, and most of them did not express CTLA-4 (Fig. 6a and b). However, numerous CTLA-4 expressing Foxp3-eGFP cells were detected in the lung after ASC sup treatment (Fig. 6d and e). CTLA-4 and Foxp3 expression was higher in the ASC sup treatment group compared to that of the control sup treatment group; almost all Foxp3-eGFP cells in the lungs of ASC sup-treated mice strongly expressed CTLA-4, a surface marker for T_reg_-cell activation (Fig. 6c-e).

### IL-10 and TGF-β were highly expressed in ASC sup

**Fig. 7:**
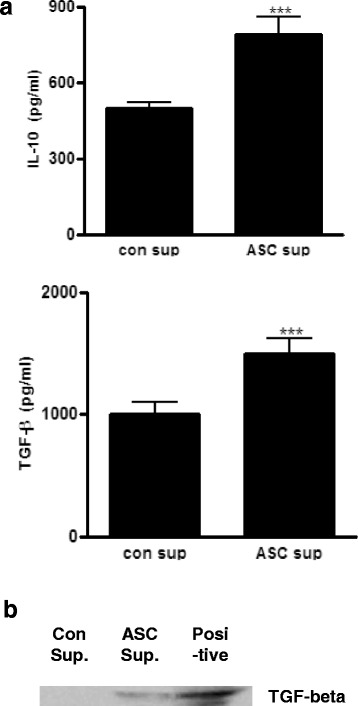
Protein analysis of con sup and ASC sup. **a** IL-10 and TGF-β expression level in ASC sup and con sup were measured by ELISA. **b** Both concentrated culture medium. 30 μg was loaded and the proteins were separated using SDS-PAGE. The proteins were transferred onto a nitrocellulose membrane and incubated with antibody specific for TGF-β. (^***^ *p* < 0.001, experiments were performed in triplicate)

To identify the proteins present in control sup and ASC sup, each sample was analyzed using SDS-PAGE. Various proteins (3–100 kDa and heavier) were observed in both samples, but extra protein bands were identified in ASC sup (Additional file 2: Figure S2). Proteins of approximately, 150 kDa, 80 kDa, and 50 kDa, as well as several proteins below 26 kDa were additionally observed in ASC sup. To determine if these small additional proteins were IL-10 and/or TGF-β, we compared IL-10 and TGF-β levels in control sup and ASC sup using ELISA. Levels of both of these T_reg_ cell-related cytokines were significantly higher in ASC sup than in control sup (Fig. 7a). In addition, high levels of TGF-β in ASC sup were demonstrated by western blot analysis using an anti-TGF-β monoclonal antibody (Fig. 7b).

## Discussion

There are many reports on the ability of stem cells to suppress allergy and immune responses, but the detailed mechanisms and core molecules have not been identified. Although mechanistically unclear, most of the evidence suggests that T_reg_ cells make important contributions to the suppression of Th2 immune responses during allergic airway inflammation [6, 14]. Herein, we demonstrated that the ASC-derived secretome, contained in the culture supernatant, even without ASCs, could ameliorate allergic airway inflammation through suppressed Th2 cytokine production and recruitment of activated T_reg_ cells into the airway.

Lin et al. observed that after transplantation of bone marrow-derived MSCs into the stomach of mice with an *H. pylori* infection, migration of MSC cells, from the subserosal to the mucosal layer of the stomach, was detected at 28 days posttransplantation [19]. The MSCs significantly stimulated systemic and local IL-10-secreting T cells and T_reg_ cells [19]. Nemeth et al. demonstrated that bone marrow-derived MSCs suppressed allergic responses in a mouse model of ragweed-induced asthma, and elicited the recruitment of T_reg_ cells to the lung and enhanced the concentration of TGF-β in serum and in BALF [20]. However, these effects were suppressed by TGF-β inhibitor treatment. Cahill et al. demonstrated that protection mediated by MSCs was associated with activated Treg in the lung and increasing IL-10 production in allergic airway inflammation [21]. In previous studies, we also found increased T_reg_ cell and IL-10-secreting T cells recruitment after ASC adaptive transfer, and the immunosuppressive ability of ASCs was closely related to prostaglandin E2 and TGF-β production [6, 14].

Although there are many reports about the immunomodulation effects of MSCs, we found only a few reports about the immunosuppressive effects of culture supernatant and core molecules from MSCs. Culture supernatant of human neural stem cells (HB1.F3) has a therapeutic effect on acute stroke and intracerebral hemorrhage, and suppresses the proliferation of human peripheral T cells, including the CD3 + CD103+ subpopulation [8]. After treating T cells with culture supernatant from human neural stem cells, the secretion of IL-2 was significantly decreased, whereas that of IL-4, IL-10, TNF-α, and IFN-γ was increased. In this study, we found amelioration of asthma signs in an OVA-Alum-induced mouse model of allergic airway inflammation through ASC culture supernatant treatment (Figs. 1 and 2). This was associated with a decrease in Th2 cytokine (IL-4, IL-5, and IL-13) production in lung and peripheral lymph node cells and an increase in IL-10 and TGF-β levels (Fig. 3). Th2 cytokine-secreting T cells in LLN were also decreased by ASC sup treatment (Fig. 4). In addition, T_reg_ cell recruitment in ACS sup treatment group was also observed (Fig. 6). Therefore, ASC sup probably has immunosuppressive ability similar to ASCs.

Lee at al. investigated the secretome of human adipose tissue-derived mesenchymal stem cells (hASCs) using liquid chromatography coupled with tandem mass spectrometry [13]. They identified about 200 individual proteins in hASC-conditioned media; among them, 118 proteins were secreted at higher levels. The secretome included a variety of cytokines and chemokines such as IL-6, IL-8, CXCL6, and monocyte chemotactic protein-1 (MCP-1). Their results were quite different from our results; IL-6, IL-8, and MCP-1 are closely associated with movement of monocytes, whereas IL-10, TGF-β, Indoleamine 2,3-dioxygenase (IDO), and PGE2 are related to T_reg_ cell activation. To obtain high concentrations of cytokines and chemokines, they stimulated hASCs with TNF-α, a well-known mediator of inflammation. Thus, they only identified pro-inflammation-related cytokines and chemokines from stem cells; this might be quite different to what is occurring in a normal culture situation or in vivo. In our study, ASC sup was obtained without any stimulation, and we detected enhanced concentrations of IL-10 and TGF-β after cultivation of ASCs (Fig. 7).

IL-10, which is referred to as the cytokine synthesis inhibitory factor, is an anti-inflammatory cytokine that is capable of inhibiting pro-inflammatory cytokine synthesis. IL-10 is generated primarily by T_reg_ cells, and has been shown to induce T_reg_ cell differentiation [22, 23]. There are many reports about IL-10 expression by various MSCs during various situations [24–27]. Yang et al. also demonstrated that splenocytes stimulated with alloantigen in the presence of MSC culture supernatant produced a significant amount of IL-10, which was attributed to an increase in the number of IL-10-secreting cells, confirmed by an ELISPOT assay [26]. They suggested that because MSCs could not suppress T cell proliferation under IL-10 blockade, MSC secreting IL-10 plays a major role in the suppression of T cell proliferation [26]. TGF-β has also been implicated in the conversion of naive CD4^+^CD25^-^ T cells into CD4^+^CD25^+^ T cells via the activity of Foxp3. TGF-β also promotes the in vivo expansion and suppressive function of CD4^+^CD25^+^ T_reg_ cells [28, 29]. In stem cells, TGF-β is one of the most important molecules for their regeneration and differentiation ability [30, 31]. To repair wounds or tissue, stem cells must receive extrinsic signals from their surrounding environment, and integrate them with intrinsic abilities, to self-renew and differentiate to ultimately produce tissues. The TGF-β superfamily constitutes integral components for the crosstalk between stem cells and their microenvironment [32]. Elevated production of IL-10 and TGF-β in vitro and in vivo in our model led us to propose that T_reg_ cells were involved in the amelioration of the inflammatory response induced by ASC sup. In addition, CTLA-4-expressing T_reg_ cells were significantly recruited through ASC sup treatment. CTLA-4 can be found on activated T_reg_ cells, which was shown to activate the transmission of immunosuppressive signals on T effector cells by interacting the T effector ligands CD80 and CD86 [33, 34]. To find other immunomodulatory molecules in ASC sup, we also tried two-dimensional analysis of ASC sup without any stimulation. However, we did not obtain conclusive results (data not shown). In the ASC culture medium, 10% FBS was present. FBS is essential for in vitro cultivation of ASCs, and contains many seroproteins (Fig. 7). To get ASC-specific proteins, the concentration of FBS in medium would have to be reduced to a minimum level (below 1%). However, in these conditions, ASCs could not survive.

The ability of stem cells to differentiate is their most powerful function from a therapeutic standpoint; however, they also have the ability to differentiate into cancer cell lines. In addition, stem cells have many limitations in therapeutic use; as of now, only the patient’s own stem cells can be used for therapy, as the immunological rejection response needs to be avoided. Presently, a method for developing stem cells suitable for a patient’s situation has not been reported. Before clinical trials are initiated, much more needs to be known about how to control stem cell proliferation, and differentiation into specific phenotypes, induce their integration into existing neural and synaptic circuits, and optimize functional recovery in animal models closely resembling the human disease [35].

## Conclusions

In this study, we found strong immune suppressive effects of culture supernatant from ASCs in an allergic airway mouse model. This supernatant has many advantages, including safety, ease of handling, ability to be stored for long periods, and usage in patients. Although we need more information about ASC sup before use in therapy, this strategy could be used to treat many immunological diseases in the near future.

## Additional files

Additional file 1: Figure S1. Mean fluorescence intensity (MFI) of CD4^+^CD25^+^Foxp3^+^ T cell in LLN of ASC sup-treated and airway inflammation-induced mice. MFI value of CD4^+^CD25^+^Foxp3^+^ markers in LLN of ASC sup-treated and asthma-induced mice have significantly higher value than those of asthma-induced mice (**, *p* < 0.001). (PPT 131 kb)
Additional file 2: Figure S2. SDS-PAGE of supernatant after ASC cultivation. Comparison of protein composition of con sup (concentrated medium for ASCs cultivation) and ASC sup (concentrated culture supernatant after ASC cultivation for 3 days) using SDS-PAGE. Thirty micrograms of each sample was loaded into an SDS-PAGE gel. After electrophoresis, the gel was stained by Coomassie Blue (*M* molecular marker, *arrow* indicated extra proteins compared to control). (PPT 370 kb)

## Abbreviations

AHR: airway hyperresponsiveness
ASC sup: supernatant of ASCs
ASCs: adipose tissue stem cells
BALF: bronchoalveolar lavage fluid
con sup: control supernatant
ELISA: enzyme-linked immunosorbent assay
FBS: fetal bovine serum
Foxp3-GFP: expressing GFP-tagged Foxp3
H&E: hematoxylin and eosin
hASCs: human adipose tissue-derived mesenchymal stem cells
IDO: indoleamine 2,3-dioxygenase
IFN-γ: interferon gamma
IL: interleukin
LLNs: lung-draining lymph nodes
LPS: lipopolysacharide
MCP-1: monocyte chemotactic protein-1
MFI: mean fluorescence intensity
MSCs: mesenchymal stem cells
PAGE: polyacrylamide gel electrophoresis
PAS: periodic acid-Schiff
PBS: phosphate-buffered saline
PenH: enhanced pause
PGE2: prostaglandin E2
TGF-β: transforming growth factor-beta
TNF-α: tumor necrosis factor-alpha
T_reg_: regulatory T cells
