# Supplementary material for: Culture supernatant of adipose stem cells can ameliorate allergic airway inflammation via recruitment of CD4+CD25+Foxp3 T cells
Source: Stem Cell Res Ther. 2017 Jan 23;8:8. doi: 10.1186/s13287-016-0462-5 (PMC5259897; doi:10.1186/s13287-016-0462-5)
Supplement: Additional file 1: Figure S1. — Mean fluorescence intensity (MFI) of CD4+CD25+Foxp3+ T cell in LLN of ASC sup-treated and airway inflammation-induced mice. MFI value of CD4+CD25+Foxp3+ markers in LLN of ASC sup-treated and asthma-induced mice have significantly higher value than those of asthma-induced mice (**, p < 0.001). (PPT 131 kb) [file 13287_2016_462_MOESM1_ESM.ppt]

## Slide 1
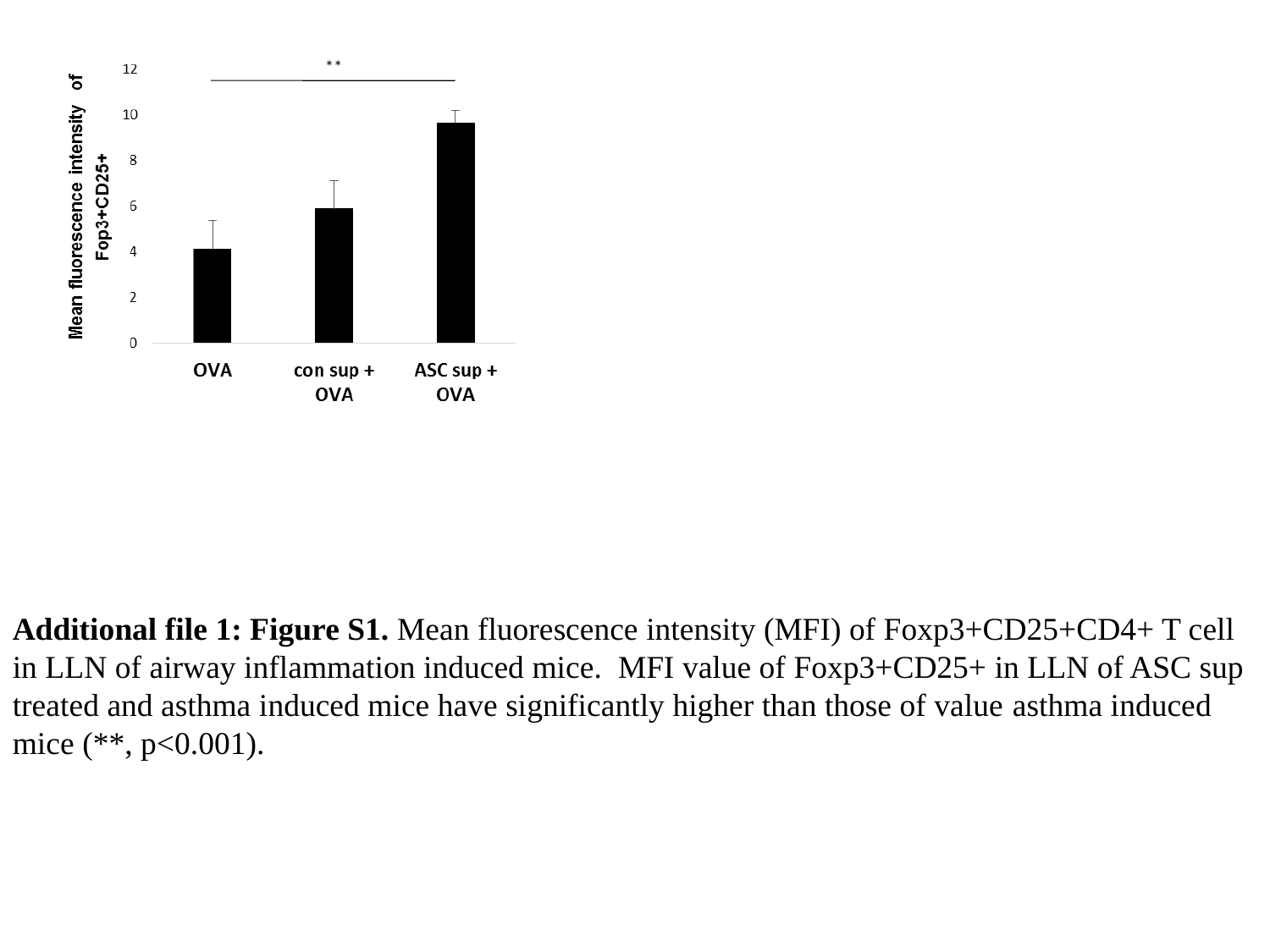

**
Additional file 1: Figure S1. Mean fluorescence intensity (MFI) of Foxp3+CD25+CD4+ T cell in LLN of airway inflammation induced mice. MFI value of Foxp3+CD25+ in LLN of ASC sup treated and asthma induced mice have significantly higher than those of value asthma induced mice (**, p<0.001).
