# Supplementary material for: Culture supernatant of adipose stem cells can ameliorate allergic airway inflammation via recruitment of CD4+CD25+Foxp3 T cells
Source: Stem Cell Res Ther. 2017 Jan 23;8:8. doi: 10.1186/s13287-016-0462-5 (PMC5259897; doi:10.1186/s13287-016-0462-5)
Supplement: Additional file 2: Figure S2. — SDS-PAGE of supernatant after ASC cultivation. Comparison of protein composition of con sup (concentrated medium for ASCs cultivation) and ASC sup (concentrated culture supernatant after ASC cultivation for 3 days) using SDS-PAGE. Thirty micrograms of each sample was loaded into an SDS-PAGE gel. After electrophoresis, the gel was stained by Coomassie Blue (M molecular marker, arrow indicated extra proteins compared to control). (PPT 370 kb) [file 13287_2016_462_MOESM2_ESM.ppt]

## Slide 1
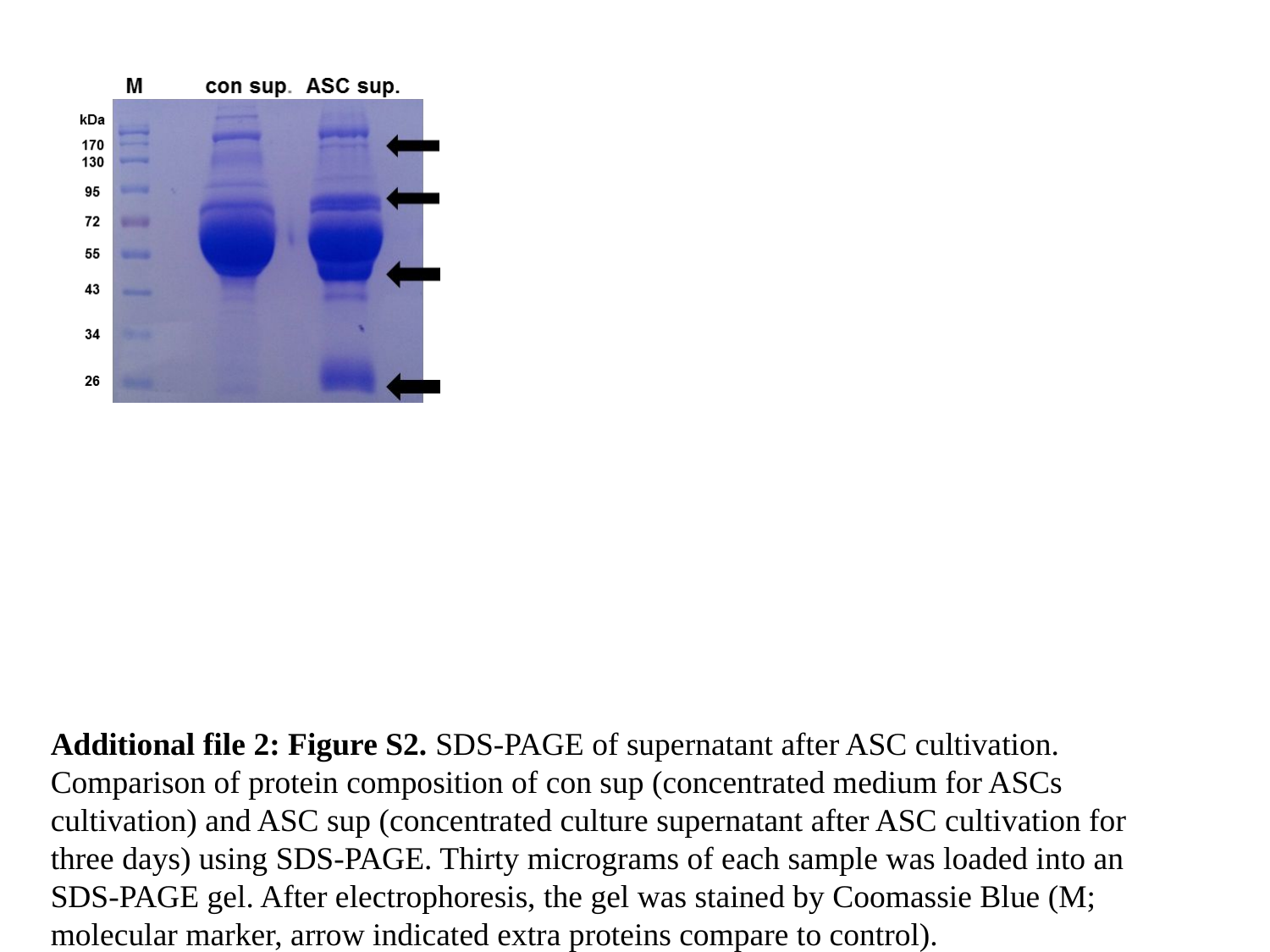

Additional file 2: Figure S2. SDS-PAGE of supernatant after ASC cultivation. Comparison of protein composition of con sup (concentrated medium for ASCs cultivation) and ASC sup (concentrated culture supernatant after ASC cultivation for three days) using SDS-PAGE. Thirty micrograms of each sample was loaded into an SDS-PAGE gel. After electrophoresis, the gel was stained by Coomassie Blue (M; molecular marker, arrow indicated extra proteins compare to control).
